# Optimized RNP transfection for highly efficient CRISPR/Cas9-mediated gene knockout in primary T cells

**DOI:** 10.1084/jem.20171626

**Published:** 2018-03-05

**Authors:** Akiko Seki, Sascha Rutz

**Affiliations:** Department of Cancer Immunology, Genentech, South San Francisco, CA

## Abstract

CRISPR (clustered, regularly interspaced, short palindromic repeats)/Cas9 (CRISPR-associated protein 9) has become the tool of choice for generating gene knockouts across a variety of species. The ability for efficient gene editing in primary T cells not only represents a valuable research tool to study gene function but also holds great promise for T cell–based immunotherapies, such as next-generation chimeric antigen receptor (CAR) T cells. Previous attempts to apply CRIPSR/Cas9 for gene editing in primary T cells have resulted in highly variable knockout efficiency and required T cell receptor (TCR) stimulation, thus largely precluding the study of genes involved in T cell activation or differentiation. Here, we describe an optimized approach for Cas9/RNP transfection of primary mouse and human T cells without TCR stimulation that results in near complete loss of target gene expression at the population level, mitigating the need for selection. We believe that this method will greatly extend the feasibly of target gene discovery and validation in primary T cells and simplify the gene editing process for next-generation immunotherapies.

## Abstract (precis)

Seki and Rutz describe an optimized Cas9/RNP transfection approach to enable highly efficient CRISPR-mediated gene knockout in primary mouse and human T cells without T cell receptor stimulation that results in near complete loss of target gene expression at the population level.

## Introduction

The broad application of CRISPR (clustered, regularly interspaced, short palindromic repeats)/Cas9 (CRISPR-associated protein 9) technology has ushered in a new era of genomic editing. Introduction of Cas9, a RNA-guided nuclease and a short guide RNA (gRNA), facilitates the generation of site-specific DNA breaks, which are repaired by cell-endogenous mechanisms. One such mechanism, mutagenic nonhomologous end-joining (NHEJ), creates insertions or deletions (InDels) at the site of the break and frequently results in loss-of-function mutations. In contrast, homologous recombination (HR), which makes use of an exogenously introduced donor template DNA, enables precise changes to a genomic sequence (Jinek et al., 2012; Cong et al., 2013; Mali et al., 2013; Hsu et al., 2014). CRISPR/Cas9 has since become the go-to approach to generate KO and knock-in mutants in a variety of species. Although the technology has been successfully applied in a multitude of cell lines, its application in primary cells is currently more limited because of difficulties in efficiently transfecting these cells. These complications are not unlike those faced previously with RNAi technology (Rutz and Scheffold, 2004; Mantei et al., 2008).

T lymphocytes are critical regulators and effectors of adaptive immune responses. The study of gene function in primary T cells is highly relevant not only from a research perspective but also for T cell–based immunotherapies (Ren and Zhao, 2017). Several strategies are being pursued to incorporate gene editing into the development of next-generation chimeric antigen receptor (CAR) T cells for the treatment of various cancers. Those approaches include the deletion of endogenous TCRs and HLA class I to generate universal allogenic “off-the-shelf” CAR T cells or the disruption of inhibitory receptors, such as CTLA-4 or PD-1 (Liu et al., 2017; Ren et al., 2017a,b; Rupp et al., 2017), and the targeting of CAR constructs to the endogenous TCR α constant locus (Eyquem et al., 2017). Target antigens recognized by CARs, such as CD7, can be knocked out on CAR T cells themselves to avoid self-elimination (Gomes-Silva et al., 2017). The prospect of novel immunotherapies has also reinvigorated research of mechanisms of T cell activation and differentiation. However, definitive assessment of gene function in this area still requires the generation of KO mice or the use of experimental cell line systems for CRISPR-mediated gene KO, such as Jurkat cells (Chi et al., 2016).

Earlier attempts to apply CRISPR/Cas9 for gene editing in primary human T cells used either viral delivery of Cas9 and gRNA (Wang et al., 2014; Li et al., 2015) or transfection by electroporation of gRNA/Cas9 expression constructs (Mandal et al., 2014; Su et al., 2016). These approaches resulted in low targeting efficiencies, and DNA electroporation proved highly toxic for T cells. More recent approaches using electroporation of Cas9 ribonucleoproteins (RNPs), complexes of recombinant Cas9 with in vitro–transcribed or synthetic single guide RNA (sgRNA), to transfect activated human T cells resulted in 50% to 90% efficiency across different targets, including CXCR4, CCR5, PD-1, and CD7 (Hendel et al., 2015; Schumann et al., 2015; Gomes-Silva et al., 2017; Ren et al., 2017a; Rupp et al., 2017). Primary mouse T cells are an essential research tool, as they enable studies of gene function ex vivo and in vivo in a highly physiologically relevant manner. The recent development of Cas9-transgeneic mice (Platt et al., 2014; Chu et al., 2016) has made it possible to subject primary T cells obtained from these mice to CRISPR/Cas9 gene editing. However, no protocols exist to date to apply Cas9/RNP-mediated gene KO with reasonable efficiency to mouse primary T cells, which would greatly expand the utility of CRISPR/Cas9 to include mouse lines of different genetic or KO background. Compared with human T cells, primary mouse T cells have proven to be more resistant to transfection and gene silencing, at least by RNAi (Mantei et al., 2008).

We have developed an optimized Cas9 RNP transfection approach that allows CRISPR/Cas9-mediated gene KO in both mouse and human T cells with high efficiency, routinely resulting in greater than 90% KO cells with a single transfection, as measured by loss of target protein expression, thus largely eliminating the need for selection and isolation of successfully targeted subpopulations for functional studies. More importantly, the protocol does not require T cells to be activated before transfection, thus enabling studies of genes involved in T cell activation and differentiation. Double KOs can be generated with similar efficiency. For proof of concept, we have successfully applied this approach to KO cell surface receptors with relevance for immunotherapies, including CXCR4, PD-1, CTLA4, and TIGIT, transcription factors such as FoxP3, and cytokines such as IFN-γ on both CD4^+^ and CD8^+^ T cells from mouse and human. We believe this approach can be easily adapted for other primary cell types.

## Results

### Retroviral expression of gRNA/Cas9 is inefficient in primary T cells

Although mouse T cells are essential research tools, successful application of CRISPR/Cas9-mediated gene editing has not yet been reported for these cells. The ability to do so would enable fast and cost-effective loss-of-function, and adoptive transfer studies, which currently require the generation of KO mice. Both lentiviral and adenoviral systems have been used to introduce Cas9 and gRNA in human T cells, generally resulting in low KO efficiency (Wang et al., 2014; Li et al., 2015). Retroviral systems can be more efficient in transducing T cells, and are being used routinely to deliver either protein-coding genes or short hairpin RNAs to T cells with reasonable efficiency. We sought to apply this approach to CRISPR/Cas9 by designing a retroviral construct that expressed a single guide RNA (sgRNA) under the control of a U6 promoter, in addition to a Cas9 gene and GFP reporter cassette expressed via the PGK promoter (Fig. S1 A). Unfortunately, but not unexpectedly given the size of the Cas9 gene, retroviral transduction of anti-CD3/anti-CD28 stimulated cultured mouse CD4^+^ T cells resulted in very low (∼5%) transduction rates, as indicated by GFP expression 48 h after infection (Fig. S1 B). Testing four sgRNAs per gene targeting either CD4 or CD90, two surface markers whose expression could be easily assessed by flow cytometry, we observed that within 72 h after infection, between 20% and 40% of GFP^+^ T cells completely lost surface expression of CD4 or CD90, respectively (Fig. S1 C). It is likely that this relatively low KO efficiency within the GFP-positive population was due to the low expression level of Cas9 on a per-cell basis, as observed by the low levels of GFP fluorescence (Fig. S1 B). These data are consistent with previously published results with viral CRISPR/Cas9 delivery systems in human T cells (Wang et al., 2014; Li et al., 2015), demonstrating that viral “all-in-one” constructs are not a viable option for high-efficiency gene targeting in primary T cells.

### Electroporation of Cas9 RNP enables efficient target gene KO in activated primary mouse T cells

**Figure 1.**
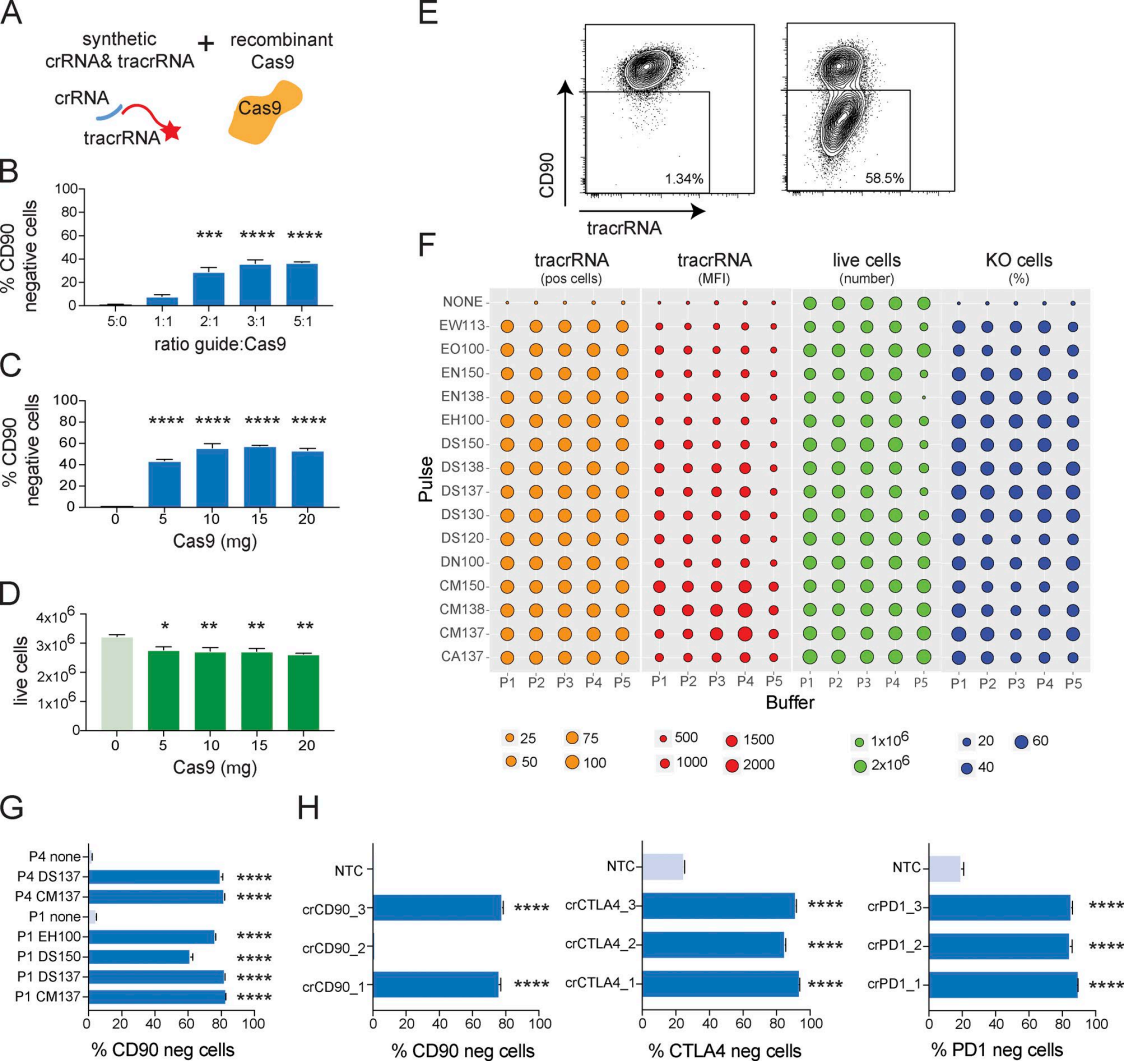
**Nucleofection of RNPs leads to highly efficient target gene KO in activated mouse T cells. (A)** Schematic depiction of RNP components, chemically stabilized crRNA, fluorescently labeled tracrRNA, and recombinant Cas9 protein. **(B)** KO efficiency as measured by CD90-negative CD8^+^ T cells 72 h after nucleofection of RNPs (DN100/P3) targeting CD90, and titration of gRNA to Cas9 ratio. Data are presented as mean ± SD (*n* = 2) and representative of two independent experiments. **(C and D)** KO efficiency as measured by CD90-negative CD8^+^ T cells (C) and cell viability 72 h after nucleofection of RNPs (DN100/P3) targeting CD90 (D), and titration Cas9 amount. Data are presented as mean ± SD (*n* = 2) and representative of two independent experiments. **(E)** Example of KO efficiency of RNP transfection targeting CD90 with 3:1 RNA/Cas9 ratio and 10 µg Cas9 3 d after transfection. **(F)** Systematic optimization of nucleofection parameters for RNP transfection of activated mouse CD8^+^ T cells. Analysis of transfection efficiency (ATTO550 expression and MFI), cell viability, and CD90 KO frequency 48 h after transfection. Data are from one experiment. **(G)** Comparison of KO efficiency by flow cytometry after RNP transfection using selected nucleofection pulses and buffers for targeting CD90 in CD8^+^ activated mouse T cells. Data are presented as mean ± SD (*n* = 2) and representative of two independent experiments. **(H)** KO efficiency as measured by flow cytometry using optimized RNP transfection in activated mouse CD8 T cells targeting CD90, CTLA4, or PD1 compared with target expression in cells transfected with NTC. Data are presented as mean ± SD (*n* = 2) and representative of three independent experiments. *, P < 0.05; **, P < 0.01; ***, P < 0.001; ****, P < 0.0001 by one-way ANOVA.

Recent studies reported successful gene editing with frequencies between 50% and 90% when transfecting preactivated human T cells with Cas9 RNPs (Hendel et al., 2015; Schumann et al., 2015; Gomes-Silva et al., 2017; Ren et al., 2017a; Rupp et al., 2017). To improve KO efficiency in mouse T cells and to eliminate the need for cloning constructs, we attempted to optimize Cas9 RNP by nucleofection using an electroporation approach that we had previously applied successfully to the transfection of siRNA oligonucleotides into primary T cells (Mantei et al., 2008). We used chemically modified synthetic target gene–specific CRISPR RNAs (crRNA) and tracer RNAs (tracrRNAs), the latter mediating the interaction with Cas9. The tracrRNA was fluorescently labeled to track transfection (Fig. 1 A). Using CD90 as a target, we first determined the amount of crRNA/tracrRNA and the ratio of gRNA to Cas9 protein required for precomplexing that resulted in maximal target gene KO. We kept the amount of Cas9 constant at 5 µg (30 pmol) for these studies. Per condition, we transfected two million mouse CD8^+^ T cells, which had been stimulated with anti-CD3/anti-CD28 for 3 d. We used the Lonza 4D nucleofection system with pulse DN-100 and buffer P3, the conditions recommended by the manufacturer for plasmid transfection of primary mouse T cells. Within the RNP complex, gRNA and Cas9 are predicted to exist in a 1:1 molar ratio. However, we found that KO efficiency, as measured by flow cytometry 3 d after transfection, was dramatically increased by providing the gRNA in a 3:1 excess, whereas a further increase in gRNA did not provide additional benefit (Fig. 1 B). We achieved a KO efficiency of ∼40% CD90-negative CD8^+^ T cells in the culture (Fig. 1 B). Next, we wanted to determine the optimal amount of recombinant Cas9. We used the same culture and transfection protocol with a 3:1 ratio of gRNA/Cas9. We found that increasing the amount of Cas9 to 10 µg (60 pmol) per transfection increased KO efficiency. Further increase was not beneficial (Fig. 1 C). The amount of Cas9 did not have any impact on cell viability, with overall viability being >80% (Fig. 1 D). This initial optimization allowed us to knock out CD90 in ∼60% of all T cells in the culture (Fig. 1 E). This efficiency was in line with previous studies in human T cells (Hendel et al., 2015; Schumann et al., 2015). Notably, we based our measure of efficiency on actual loss of target protein expression, whereas previous studies determined gene editing rates by quantification of InDel frequencies.

We reasoned that the nucleofection protocol, which was tailored for transfecting plasmid DNA, might not be optimal for RNP transfection, given that the physicochemical properties of RNPs are very different. Using a systematic optimization approach that tested five different buffers and 15 different electroporation pulses, we evaluated transfection efficiency (ATTO550 fluorescently labeled tracrRNA mean fluorescence intensity [MFI]), cell viability (number of live cells in the culture), and, most importantly, KO cell frequency (CD90-negative cells by flow cytometry) 2 d after transfection (Fig. 1 F). To sensitize our assay to differences in KO frequency arising from transfection efficiency, we used a suboptimal amount of 30 pmol Cas9 per transfection. Using this empirical approach, we identified conditions that not only further improved cell viability but also drastically increased the frequency of KO T cells. We selected a panel of six conditions and confirmed KO efficiency with optimal Cas9 amount 3 d after transfection, targeting CD90 (Fig. 1 G). Based on these studies, we selected CM137/P4 as the optimal nucleofection condition for CRISPR/Cas9 KO in preactivated mouse T cells, as it represented the best combination of cell viability and KO efficiency. We went on to apply this protocol to target CD90, CTLA-4, and PD-1 in activated CD8^+^ T cells, testing three crRNAs per target. To assess CTLA-4 and PD-1 expression, T cells were restimulated for 48 h after a 2-d resting period. Approximately 80% of T cells that had been transfected with a nontargeting control Cas9 RNP (NTC) up-regulated CTLA-4 and PD-1 expression, demonstrating that the cells were viable and responsive to stimulation. Importantly, we obtained greater than 80% KO efficiency for every target with most of the crRNAs tested (Fig. 1 H).

### RNP containing in vitro–transcribed sgRNA enable efficient target gene KO in primary T cells

Although the use of synthetic crRNA–tracrRNA pairs has many advantages, a sgRNA can be synthesized (Hendel et al., 2015) or generated by in vitro transcription from PCR productions containing a T7 promoter (Schumann et al., 2015). The latter approach, although more labor intensive, can be more economical. We wanted to determine how in vitro transcribed sgRNA compared in terms of KO efficiency. sgRNAs were precomplexed with recombinant Cas9 and introduced into target cells by nucleofection (Fig. S2 A). We first reevaluated the ratio of gRNA to Cas9, and consistent with our data using synthetic guides, a 3:1 RNA/Cas9 ratio resulted in the highest KO efficiency (Fig. S2 B). We tested this approach using the optimal amount of Cas9 (10 µg/60 pmol) and targeted CD90, PD-1, and CTLA-4 in cultured mouse CD8^+^ T cells that had been stimulated with anti-CD3/anti-CD28 for 3 d using two sgRNAs per target. At least one guide resulted in ∼90% KO efficiency in each case (Fig. S2 C). We also wanted to determined how the cell number per transfection affected KO efficiency. Interestingly, between one and ten million T cells could be transfected without any loss in KO efficiency (Fig. S2 D). Collectively, we found that nucleofection of Cas9 RNPs with either sgRNAs or crRNA–tracrRNA pairs led to highly efficient (80–90%) target gene KO in activated mouse T cells.

### Efficient CRISPR/Cas9-mediated gene KO in nonactivated human T cells

**Figure 2.**
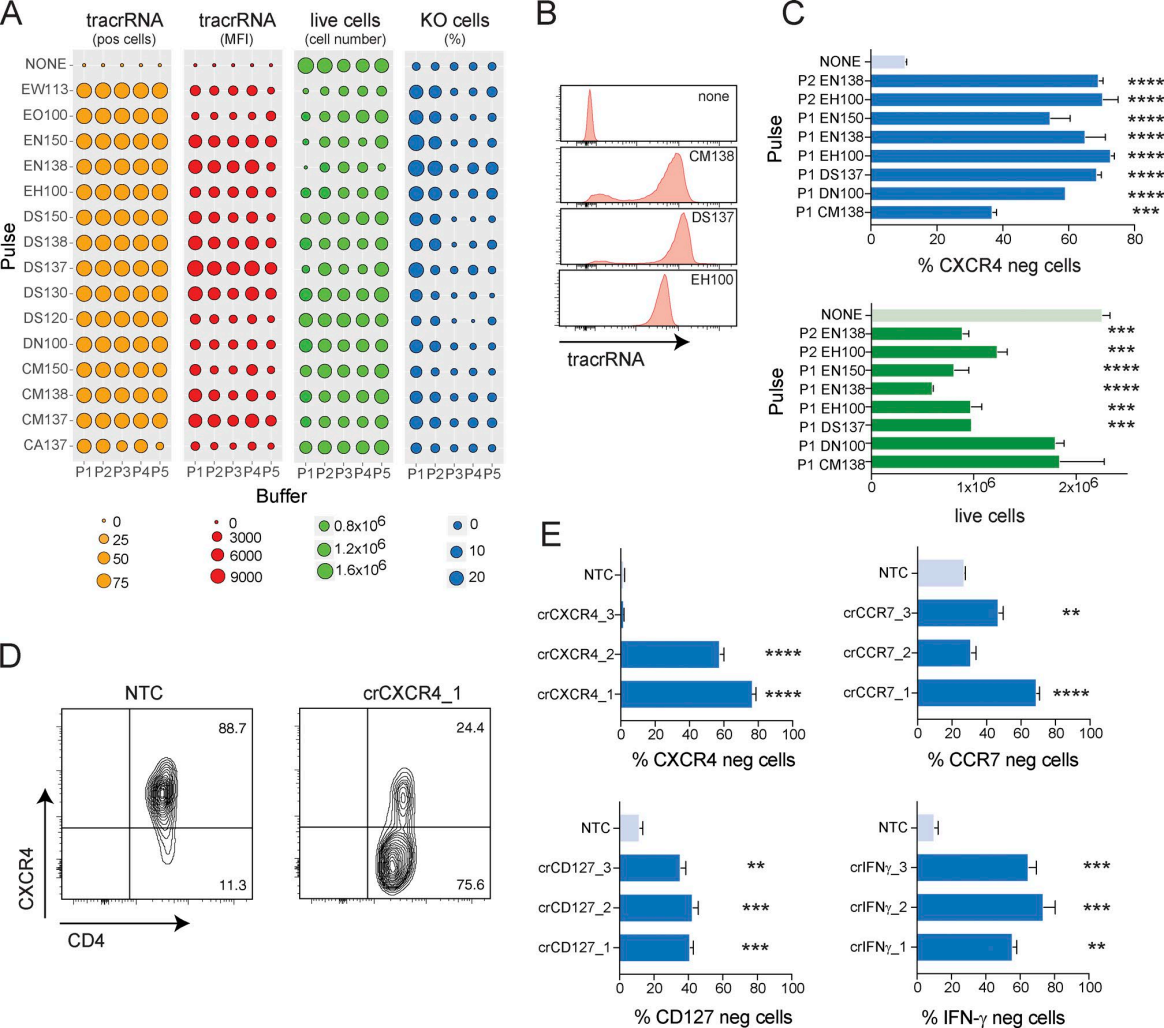
**Nucleofection of RNPs leads to highly efficient target gene KO in nonactivated human T cells. (A)** Systematic optimization of nucleofection parameters for RNP transfection of nonactivated human CD4^+^ T cells. Analysis of transfection efficiency (ATTO550 expression and MFI), cell viability, and CXCR4 KO frequency 48 h after transfection. Data are from one experiment. **(B)** Examples of transfection efficiency (ATTO550-labeled tracrRNA) with different nucleofection pulses 48 h after transfection (gated on live cells). **(C)** Comparison of KO efficiency and cell viability by flow cytometry 72 h after RNP transfection using selected nucleofection pulses and buffers for targeting CXCR4 in nonactivated human CD4^+^ T cells. Data are presented as mean ± SD (*n* = 2) and representative of two independent experiments. **(D)** CXCR4 expression by flow cytometry 72 h after RNP transfection using the EH100/P2 condition. **(E)** KO efficiency as measured by flow cytometry using optimized RNP transfection in nonactivated human CD4^+^ T cells targeting CXCR4, CCR7, CD127, or IFN-γ compared with target expression in cells transfected with NTC. Data are presented as mean ± SD (*n* = 2) and representative of two independent experiments. **, P < 0.01; ***, P < 0.001; ****, P < 0.0001 by one-way ANOVA.

Previous experience with siRNA-mediated gene knockdown in primary T cells (Mantei et al., 2008) suggested that the chemical stabilization of synthetic crRNA/tracrRNA would enable efficient gene KO in resting nonactivated T cells. It is important to keep in mind that to assess genes involved in cell activation or differentiation, target gene KO has to be completed before activating the cells through TCR stimulation. Depending on the half-life of the protein, loss of target protein expression may take 48–72 h or longer. Human T cells can be cultured in complete T cell media for several days without the need for TCR or cytokine stimulation. We hypothesized that a systematic optimization of the transfection conditions would allow us to significantly improve upon existing protocols. To this end, we isolated CD4^+^ T cells from human peripheral blood mononuclear cells (PBMCs) and transfected them with suboptimal amounts of Cas9 with fluorescently labeled RNPs targeting the chemokine receptor CXCR4. After 48 h, we analyzed transfection efficiency, cell viability, and KO efficiency across our optimization panel (Fig. 2 A). Similar to mouse T cells, transfection efficiency varied depending on the pulse (Fig. 2 B), but not the buffer conditions, whereas viability was dependent on both parameters (Fig. 2 A). We identified several conditions that resulted in reasonable KO efficiency and went on to confirm these with optimal Cas9 amount and a 3-d incubation (Fig. 2 C). We identified EH100/buffer P2 as the best combination of KO efficiency and cell viability for resting human T cells, resulting in ∼75% KO efficiency and 60% cell viability (Fig. 2, C and D). We applied this condition to KO CCR7, CD127, and IFN-γ in addition to CXCR4 using three guides per target gene. To assess IFN-γ expression, transfected T cells were cultured under Th1-polarizing conditions for 3 d and restimulated with PMA/ionomycin. Approximately 90% of T cells that had been transfected with nontargeting control RNP expressed IFN-γ (Fig. 2 E), demonstrating that the cells were viable and fully responsive to stimulation. Across all four targets, we observed between 40% and 80% KO efficiency, depending on the crRNA (Fig. 2 E).

We next tested CRISPR/Cas9-mediated gene KO in activated human T cells. We used the same conditions identified for resting T cells and transfected in vitro–expanded Foxp3-positive regulatory T cells with crRNAs targeting Foxp3. 2 d after transfection, we assessed Foxp3 expression and observed between 25% and 85% Foxp3-negative T cells, depending on the crRNA (Fig. S3). Collectively, we identified conditions that allowed for efficient target gene KO in both resting and activated human T cells.

### CRISPR/Cas9-mediated gene KO in nonactivated mouse T cells

**Figure 3.**
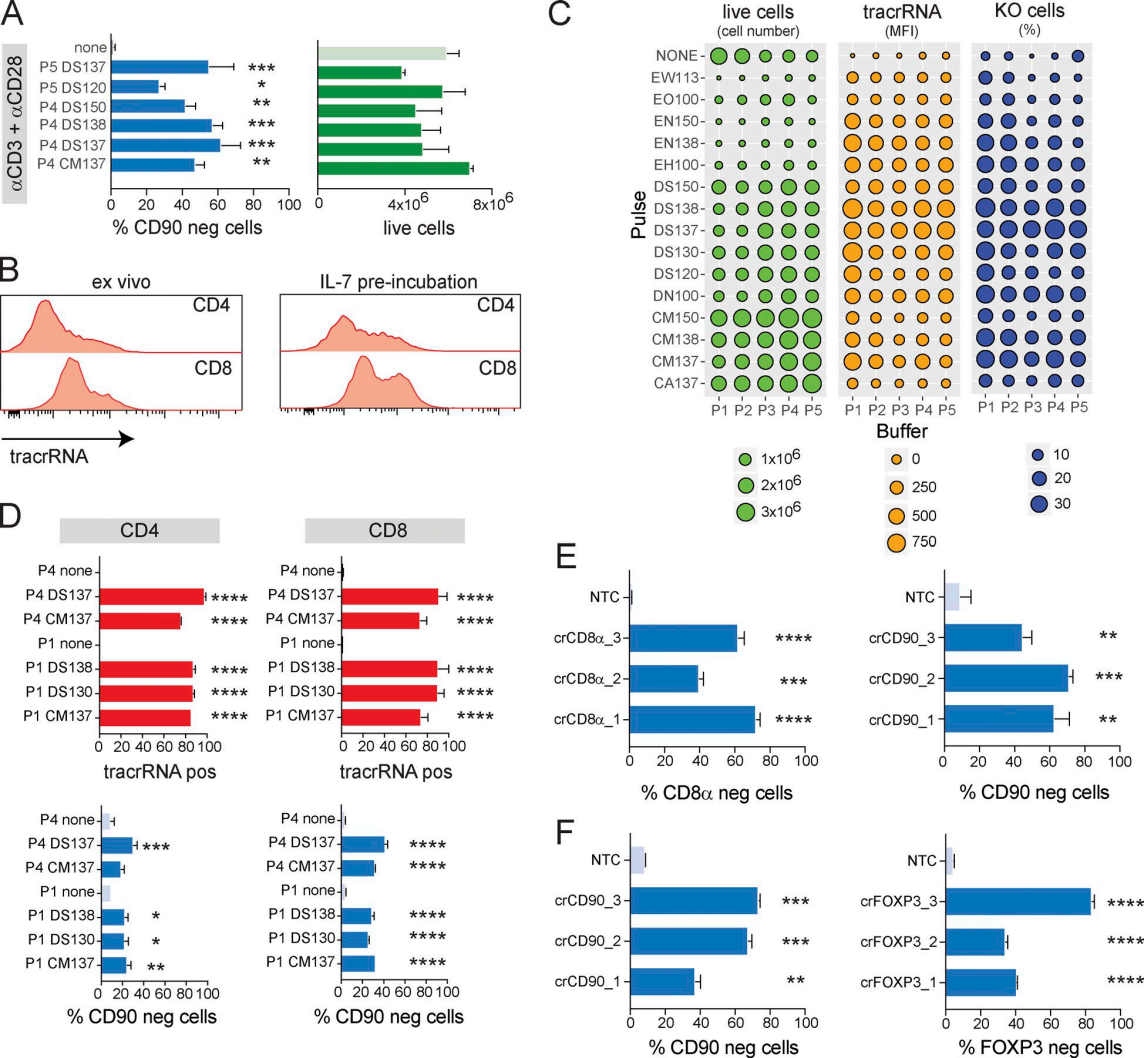
**RNP transfection enables gene KO before TCR stimulation in nonactivated mouse T cells. (A)** Comparison of KO efficiency and cell viability by flow cytometry 48 h after RNP transfection using selected nucleofection pulses and buffers for targeting CD90 in mouse CD8^+^ T cells transfected before anti-CD3/anti-CD28 stimulation. Data are presented as mean ± SD (*n* = 2) and representative of two independent experiments. **(B)** Transfection efficiency (ATTO550-labeled tracrRNA) 48 h after transfection of mouse CD4^+^ or CD8^+^ T cells transfected ex vivo or after 24-h preincubation with IL-7 (gated on live cells). **(C)** Systematic optimization of nucleofection parameters for RNP transfection of IL-7–preincubated nonactivated mouse CD8^+^ T cells. Analysis of cell viability, transfection efficiency (ATTO550 MFI), and CD90 KO frequency 72 h after transfection. Data are from one experiment. **(D)** Comparison of transfection and KO efficiencies by flow cytometry 72 h after RNP transfection using selected nucleofection pulses and buffers for targeting CD90 in mouse CD4^+^ or CD8^+^ T cells cultured with IL-7. Data are presented as mean ± SD (*n* = 2) and representative of two independent experiments. **(E and F)** KO efficiency as measured by flow cytometry using optimized RNP transfection in nonactivated mouse CD8^+^ T cells targeting CD8 or CD90 (E) and nonactivated mouse CD4^+^ T cells targeting CD90 or Foxp3 compared with NTC (F). Data are presented as mean ± SD (*n* = 2) and representative of two independent experiments. *, P < 0.05; **, P < 0.01; ***, P < 0.001; ****, P < 0.0001 by one-way ANOVA.

We next wanted to establish CRISPR/Cas9-mediated gene KO in resting mouse T cells, which has not been previously reported. Primary mouse T cells are much more difficult to culture in the absence of TCR stimulation than human T cells, as they usually die within 24–48 h (Vella et al., 1997; Kishimoto and Sprent, 1999). We first optimized the nucleofection conditions for resting mouse CD8^+^ T cells targeting CD90 (Fig. S4, A–C). After a resting period of 2 h, T cells were either left untreated or activated with polyclonal TCR stimulation to better understand the transfection requirements of nonactivated T cells. As expected, cell viability was low after 48 h of culture in cells that had been left unstimulated, and electroporation resulted in further reduced numbers of viable cells. In contrast, TCR stimulation strongly improved cell viability and limited the negative impact of the electroporation (Fig. S4, A–C). Transfection efficiency, as indicated by ATTO550-labeled tracrRNA fluorescence, was somewhat lower for some of the conditions tested compared with preactivated T cells. Strikingly, 48 h after transfection, we observed pronounced target gene KO in T cells that had been TCR stimulated shortly after transfection, whereas there was essentially no detectable KO in nonstimulated T cells, despite similar transfection rates (Fig. S4, A–C). We picked the top six nucleofection conditions and identified DS137/P4 as the optimal condition for RNP nucleofection of nonactivated mouse T cells with subsequent TCR stimulation, resulting in ∼60% KO efficiency and viability (Fig. 3 A). However, this protocol did not allow studies of gene function during T cell activation, as residual target protein was still present at the time of TCR stimulation.

To facilitate KO studies in non–TCR-stimulated T cells, we wanted to improve T cell viability and KO efficiency in the absence of stimulation. We cultured mouse CD4^+^ or CD8^+^ T cells with various concentrations of IL-2, IL-7, or IL-15 (Vella et al., 1997; Kishimoto and Sprent, 1999; Tan et al., 2001) for 3 d and measured cell viability as well as activation markers, such as CD69 and CD25 (Fig. S4, D and E). We found that IL-7 drastically improved cell viability in both CD4^+^ and CD8^+^ T cells without inducing proliferation or strong up-regulation of activation markers. In contrast, IL-2 and IL-15 induced proliferation in CD8^+^ T cells but did not preserve cell viability in CD4^+^ T cells (Fig. S4, D and E). We also found that a 24-h preincubation with IL-7 increased “transfectability” of CD4^+^ and CD8^+^ mouse T cells, as indicated by higher intracellular fluorescence of ATTO550-labeled tracrRNA levels (Fig. 3 B).

Having optimized TCR-independent T cell culture conditions, we next wanted to optimize the transfection conditions for nonactivated IL-7 preconditioned mouse T cells. Transfected T cells were incubated for 3 d before analyzing transfection and KO efficiency, as well as cell viability (Fig. 3 C). We tested five conditions with optimal Cas9 concentrations in both CD4^+^ and CD8^+^ T cells and again identified DS137/P4 as optimal, yielding 30–40% KO cells with 60% overall cell viability (Fig. 3, C and D). We also observed that cells displayed reduced surface expression of CD90 still maintained some level of expression after 3 d of incubation (not depicted), suggesting that protein turnover was much slower in nonactivated T cells than TCR-stimulated T cells. We therefore switched to a 5-d IL-7 incubation period before using mouse T cells for any downstream studies. This prolonged culture period did not affect overall cell viability.

Using three gRNA each, we went on to knock out CD8 and CD90 in nonactivated mouse CD8^+^ T cells (Fig. 3 E). We also knocked out CD90 and FoxP3 in nonactivated CD4^+^ T cells. To induce Foxp3 expression, CD4^+^ T cells were TCR stimulated and polarized toward iTreg cells with IL-2 and TGF-β after a 5-d incubation in IL-7 (Fig. 3 F). It is noteworthy that ∼95% of iTreg cells that had been transfected with nontargeting control RNP up-regulated Foxp3 expression (Fig. 3 F), again demonstrating that subsequent to the transfection procedure cells were fully functional and responsive to stimulation. With these conditions, we were able to achieve between 40% and 80% KO efficiency depending on the gRNA.

Our data demonstrated that it is indeed possible to induce efficient target gene KO in nonactivated mouse T cells before subjecting them to TCR stimulation.

### Further improvements to increase KO efficiency

Although we achieved up to 80% target gene KO across several targets in both mouse and human T cells, the approach did not result in complete KO at the population level. Depending on the expected phenotype and the frequency of KO cells within the population, this limitation might still require the identification of KO cells by flow cytometry, as selection of KO cells is not feasible. It is also worth noting that KO efficiencies obtained with individual guides varied greatly, suggesting that improved guide design algorithms may help to further boost KO efficiency.

**Figure 4.**
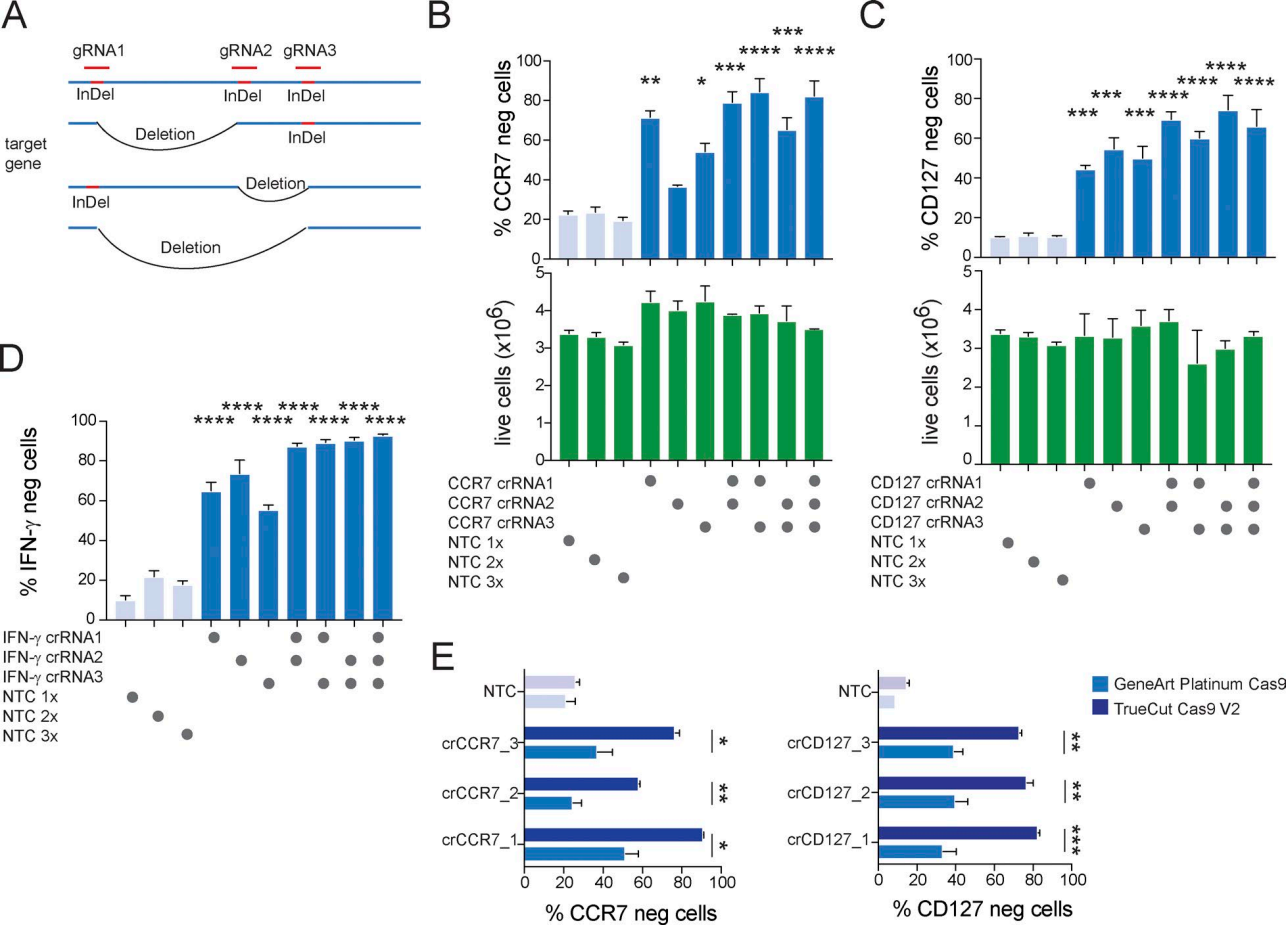
**Combination of multiple gRNAs further increases target gene KO efficiency. (A)** Schematic depiction of potential outcome of CRISPR targeting of three regions in the same gene leading either to local InDels or deletion of flanked DNA. **(B–D)** KO efficiency of targeting CCR7 (B), CD127 (C), or IFN-γ (D) using individual gRNAs or the combination of two or three gRNAs per target in nonactivated human CD4^+^ T cells 72 h after RNP transfection. Data are presented as mean ± SD (*n* = 2; one-way ANOVA) and representative of two independent experiments. **(E)** Comparison of KO efficiency 72 h after RNP transfection targeting CCR7 or CD127 in nonactivated human CD4^+^ T cells using either recombinant GeneArt Platinum Cas9 or TrueCut Cas9 V2. Data are presented as mean ± SD (*n* = 2; unpaired Student’s *t* test) and representative of two independent experiments. *, P < 0.05; **, P < 0.01; ***, P < 0.001; ****, P < 0.0001.

A single guide induces random InDel mutations in a single site, which may or may not result in loss of protein expression. Combined transfection of multiple guides per target would likely increase the chances of losing protein expression, as gene editing will occur in multiple regions of the gene and in addition to InDel mutations, deletions of regions targeted by two guides are also possible (Fig. 4 A). This approach has the added benefit of alleviating the need for guide selection. We used human CD4^+^ T cells isolated from PBMCs and transfected them with either individual guides or all possible combinations for combining up to three guides against the same target in one transfection. We targeted CCR7, CD127, and IFN-γ in this way. Again, assessing IFN-γ expression required incubation of the cells under Th1 polarizing conditions and restimulation with PMA/ionomycin. We found that in all cases, combining two or three guides led to increased KO efficiency as compared with targeting with single guides (Fig. 4, B–D). The up to threefold increase in total RNP amount per transfection did not further impact cell viability (Fig. 4, B and C, bottom) or the ability of the T cells to induce IFN-γ expression upon stimulation (Fig. 4 D, NTC conditions).

When optimizing nucleofection conditions using fluorescently labeled RNPs, we observed that transfection efficiency (i.e., MFI) did not translate directly into KO efficiency, even with highly active guides. One possible explanation was a suboptimal nuclear RNP delivery. When we compared RNPs containing GeneArt Platinum Cas9 (ThermoFisher) to RNPs containing TrueCut Cas9 V2 (ThermoFisher), we observed a strong increase in KO efficiency. In some cases, previously inactive guides showed considerable activity (Fig. 4 E). We therefore routinely used a combination of three guides per target and TrueCut Cas9 V2 for all further studies.

**Figure 5.**
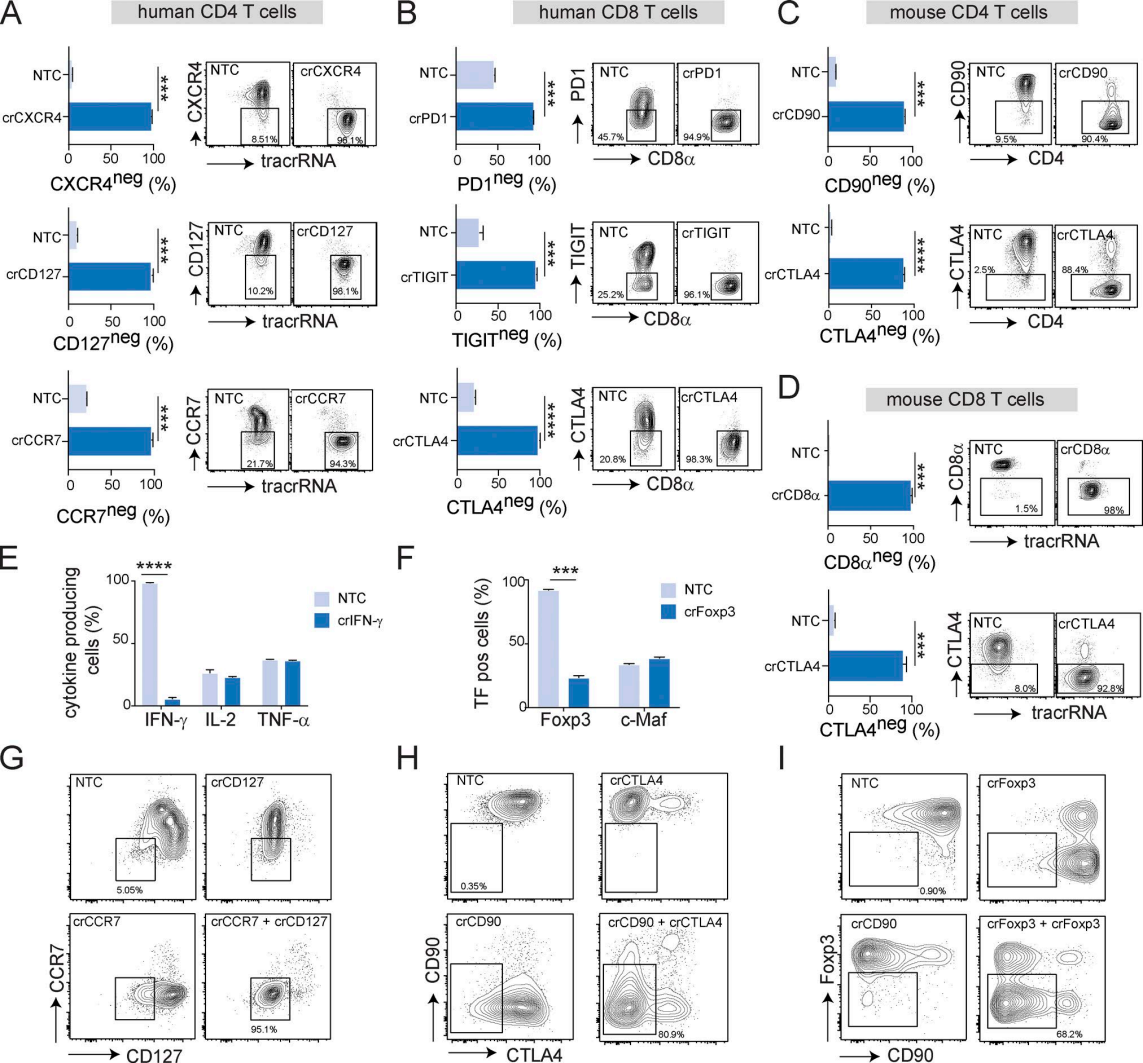
**Highly efficient target gene KO in nonactivated human and mouse primary T cells with an optimized CRISPR/Cas9 RNP transfection approach. (A–D)** KO efficiency by flow cytometry of CXCR4, CD127, and CCR7 in nonactivated human CD4 T cells 72 h after transfection (A); PD1, TIGIT, and CTLA4 in nonactivated human CD8 T cells 72 h after transfection followed by 72 h of stimulation with anti-CD3/anti-CD28 (B); CD90 and CTLA4 in IL-7–preconditioned nonactivated mouse CD4 T cells 5 d after transfection and incubation with IL-7 (C); and CD8α and CTLA4 in IL-7–preconditioned nonactivated mouse CD8^+^ T cells after transfection and 5 d of incubation in the presence of IL-7 followed by 48 h stimulation with anti-CD3/anti-CD28 (D; all compared with nontargeting control Cas9 RNP-transfected T cells). Data are presented as mean ± SD (*n* = 2) and representative of three (A) or two (B–D) independent experiments. **(E)** IFN-γ expression by flow cytometry in crIFNγ or NTC-transfected nonactivated human CD8^+^ T cells cultured for 3 d and restimulated for 4 h with PMA/ionomycin. Data are presented as mean ± SD (*n* = 2) and representative of two independent experiments. **(F)** FoxP3 and c-Maf expression by flow cytometry in crFoxp3 or NTC transfected IL-7–preconditioned nonactivated mouse CD4^+^ T cells 5 d after transfection with IL-7 followed by 3 d of polarization into iTreg with TGF-β and IL-2. Data are presented as mean ± SD (*n* = 2) and representative of two independent experiments. **(G–I)** KO efficiency by flow cytometry of double KOs targeting CD127 and CCR7 in nonactivated human CD4^+^ T cells 72 h after transfection (G), CD90 and CTLA4 in nonactivated mouse CD8 T cells after transfection and 5 d of incubation in the presence of IL-7 followed by 48 h stimulation with anti-CD3/anti-CD28 (H), or CD90 and FoxP3 in nonactivated mouse CD4^+^ T cells after transfection and 5 d of incubation with IL-7 followed by 3 d of polarization into iTreg with TGF-β and IL-2 with anti-CD3/anti-CD28 (I). Data are presented as mean ± SD (*n* = 2) and representative of two independent experiments. ***, P < 0.001; **** P < 0.0001 by unpaired Student’s *t* test.

Finally, we applied our optimized protocol for Cas9 RNP transfection of three guides per target (Table 1) to nonactivated human CD4^+^ T cells targeting CXCR4, CD127, and CCR7 (Fig. 5 A); human CD8^+^ T cells targeting PD-1, TIGIT, and CTLA4 (Fig. 5 B); mouse CD4^+^ T cells targeting CD90 and CTLA4 (Fig. 5 C); and mouse CD8^+^ T cells targeting CD8α and CTLA4 (Fig. 5 D). Across all targets, KO efficiency far exceeded previous results, reaching 85% to 98% of all cells (Fig. 5, A–D); thus enabling, for the first time, meaningful CRIPSR/Cas9 gene targeting in nonactivated T cells. Control transfected human and mouse T cells uniformly up-regulated CTLA4 expression and also induced PD-1 and TIGIT after TCR stimulation (Fig. 5, B–D), again demonstrating that the procedure did not impact their ability to be activated. To further demonstrate that KO T cells were functional, we knocked out IFN-γ in nonactivated human CD8^+^ T cells and stimulated the cells with PMA/ionomycin 3 d later. Essentially, all NTC-transfected cells were positive for IFN-γ by intracellular staining. In contrast, specific targeting of the *IFNG* locus resulted in ∼95% KO efficiency. Importantly, control and IFN-γ KO T cells produced similar levels of IL-2 and TNF-α (Fig. 5 E). Similarly, NTC-transfected mouse CD4^+^ T cells could be efficiently polarized into iTreg cells and uniformly expressed Foxp3 after 3 d of culture (anti-CD3/anti-CD28 stimulation with TGF-β and IL-2), whereas 80% of specifically targeted T cells had lost Foxp3 expression (Fig. 5 F). Both populations similarly induced TGF-β–dependent c-Maf expression (Fig. 5 F). By simply combining guides against two different targets, it was possible to generate double-KO cells with similar efficiencies and a high degree of reproducibility (Fig. 5, G–I).

**Table 1.** Summary of optimal CRISPR/Cas9 gene KO conditions (CD4^+^ and CD8^+^ T cells)

Parameter	Mouse (CD4/CD8)		Human (CD4/CD8)	
	Resting	Activated	Resting	Activated
Primary cell nucleofection solution	P4	P4	P2	P2
Pulse	DS137	CM137	EH100	EH100
Culture condition	Complete T cell media with IL-7 (preculture 24 h)	Complete T cell media with anti-CD3, anti-CD28, and IL-2	Complete T cell media	Complete T cell media with anti-CD3, anti-CD28, and IL-2
No. of cells per well	1–10 × 10^6^	1–10 × 10^6^	1–10 × 10^6^	1–10 × 10^6^

## Discussion

Here, we provide an optimized protocol for highly efficient CRISPR/Cas9-mediated gene KO in primary mouse and human T cells. The use of Cas9 RNP transfection eliminates the need for cloning and viral transduction and does not require TCR stimulation, thus allowing functional studies of genes involved in T cell activation and differentiation. The high KO efficiency that is achieved with this approach largely eliminates the need for further selection. It should be easily possible to further increase the number of genes targeted simultaneously. The ability to target multiple genes at once not only allows researchers to study combination effects but also to address redundancies within gene families.

The method is immediately amenable to functional arrayed CRISPR screens using physiologically relevant endpoints, such as cytokine production or pathway activity. Although not tested here, an alternative approach that uses retro- or lentiviral constructs to express the gRNA combined with transient transfection of Cas9 protein or mRNA by nucleofection using the conditions identified here is likely to overcome the limitations of all-in-one viral constructs and could be useful in particular for pooled CRISPR screens in primary T cells using commercially available lentiviral gRNA libraries.

Our data demonstrate that optimal transfection conditions are highly cell type–specific and may vary even among closely related cells, such as mouse versus human T cells or resting versus activated T cells. The systematic optimization of all relevant parameters of cell culture and transfection as outlined here can be a guideline for other types of primary (immune) cells, which will enable the study of gene function using CRISPR/Cas9 in more physiologically relevant systems.

## Material and methods

### Mice

All mice used in this study were housed and maintained at Genentech in accordance with American Association of Laboratory Animal Care guidelines. All experimental studies were conducted under protocols approved by the Institutional Animal Care and Use Committee of Genentech Lab Animal Research in an AAALACi-accredited facility in accordance with the Guide for the Care and Use of Laboratory Animals and applicable laws and regulations. Female C57BL/6 mice were obtained from Jackson Laboratory and were used for studies between 6 and 10 wk of age.

### PBMCs

Human PBMCs were isolated by Ficoll gradient centrifugation from buffy coats from healthy donors, collected as part of the Genentech blood donor program with written informed consent, and approval from the Western Institutional Review Board.

### Antibodies

The following antibodies were used for flow cytometry. For mouse cells, CD4 (RM4-5; eBioscience), CD8a (53-6.7; BD Biosciences), CD90.2 (53-2.1, eBioscience), PD-1 (29F.1A12; BioLegend), CTLA-4 (UC10-4B9, eBioscience), CD25 (PC61; BD Biosciences), CD69 (H1.2F3; BD Biosciences), Foxp3 (FJK-16s; eBioscience), and c-Maf (sym0F1, eBioscience) were used. For human cells, CD4 (OKT4; BioLegend), CXCR4 (12G5; eBioscience), CCR7 (G043H7; BioLegend), CD127 (A019D5; BioLegend), IFN-γ (4S.B3; eBioscience), Foxp3 (236A/E7; eBioscience) CD8 (PRA-T8, BD Biosciences), CTLA4 (BNI3; BioLegend), TIGIT (MBSA43; eBioscience), CD45 (HI30, BD Biosciences), and PD-1 (MIH4; eBioscience) were used.

### Plasmids

To obtain the all-in-one retroviral sgRNA/Cas9 construct, Cas9 fused via a self-cleaving P2A peptide to GFP was cloned into the pQCXIX vector containing a PGK promoter. A murine U6 promoter driving expression of an sgRNA was cloned in front of the PGK_Cas9_P2A_GFP expression cassette.

### Retroviral transduction

HEK293T cells were transiently transfected using FUGENE 6 with retroviral expression plasmids together with the retroviral packaging plasmids pCMV-Eco/pCMV-Gag-Pol. Viral supernatant was harvested after 48 h, filtered through a 0.45-µm filter, supplied with 10 mM Hepes, pH 7.2, and 10 µg/ml polybrene (EMD Millipore), and added to T cells that had been stimulated for 24 h. T cells (2.5 × 10^6^) were incubated in 12-well plates with 1.5 ml viral supernatant and centrifuged for 75 min at 700 *g* at 32°C. Thereafter, viral supernatant was replaced with conditioned culture medium.

### In vitro transcription

A 0.3-µM target oligonucleotide mix working solution was prepared by diluting 1 µl of each target oligonucleotide stock solution (10 µM) in 98 µl nuclease-free water. Using the GeneArt Precision gRNA Synthesis kit, a PCR assembly reaction in a 25-µl volume was set up by adding the reaction components in the order given: 12.5 µl Pfusion High-Fidelity PCR Master Mix (2×), 1 µl Tracr Fragment + T7 Primer Mix, 1 µl 0.3 µM Target F1/R1 oligonucleotide mix, and 10.5 µl nuclease-free water. The PCR cycles were as follows: initial denaturation 98°C for 10 s, 32 cycles of denaturation at 98°C for 5 s, annealing at 55°C for 15 s, and a final extension at 72°C for 1 min. PCR product was purified from an agarose gel. 16–24 ng for PCR product was used in the IVT reaction (MegaShortScript T7 kit) according to the manufacturer’s protocol and incubated at 37°C for 4 h. To remove the DNA template, 1 µl TURBO DNase was added to the reaction and the incubation continued at 37°C for 15 min. sgRNA was purified using the GeneArt Precision gRNA Synthesis kit according to the manufacturer’s protocol and eluted in 40 µl RNase-free water.

### crRNA selection

Routinely, three crRNAs were selected per target using the DESKGEN (www.deskgen.com) online platform. The target area was limited to the first ∼40% of the coding sequence, and preference was given to guides targeting different regions within this area. Guides with the highest on-target and off-target scores were selected. crRNAs were ordered from Integrated DNA Technologies (www.idtdna.com/CRISPR-Cas9) in their proprietary Alt-R format (Table S1).

### T cell purification

Murine CD4^+^ or CD8^+^ T cells were isolated from single-cell suspensions prepared from spleens and lymph nodes of C57BL/6 mice. Dead cells were removed using the Dead Cell Removal kit (catalog number 130-090-101; Miltenyi Biotec), followed by T cell isolation using the Mouse CD4^+^ T Cell Isolation kit (catalog number 130-104-454; Miltenyi Biotec) or the Mouse CD8^+^ T Cell Isolation kit (catalog number 130-104-075; Miltenyi Biotec) according to the manufacturer’s protocol. Human CD4^+^ or CD8^+^ T cells were isolated from PBMCs using the Human CD4^+^ T Cell Isolation kit (catalog number 130-096-533; Miltenyi Biotec) or the Human CD8^+^ T Cell Isolation kit (catalog number 130-096-495; Miltenyi Biotec) according to the manufacturer’s protocol. Purities were >95%. Human regulatory T cells were isolated from PBMCs using the Human CD4^+^CD25^+^CD127^dim/−^ Regulatory T Cell Isolation kit II (catalog number 130-094-775; Miltenyi Biotec) according to the manufacturer’s protocol. Purity was >90%.

### T cell cultures

All T cells were cultured in complete T cell media/RPMI media with 10% FCS (HyClone), 2 mM l-alanyl-l-glutamine (GlutaMAX; Gibco), 1 mM sodium pyruvate, 0.1 mM nonessential amino acids, 55 µM β-mercaptoethanol, 100 U/ml penicillin, 100 µg/ml streptomycin, and 10 mM Hepes (Invitrogen).

### Optimized Cas9/RNP nucleofection

#### Preparation of cells

Cells were isolated as described above.

Resting murine CD4^+^ and CD8^+^ T cells were preincubated in complete T cell media containing 5 ng/ml recombinant mouse IL-7 (catalog number 407-ML-005; R&D Systems) for 24 h before transfection.

Preactivated murine CD4^+^ and CD8^+^ T cells were cultured at 1 × 10^6^ cells/ml in complete T cell media with 5 µg/ml plate-bound anti-CD3 (clone 145-2C11; BD Biosciences) and 1 µg/ml soluble anti-CD28 (clone 37.51). For CD8^+^ murine T cells 10 ng/ml recombinant IL-2 (catalog number 402-ML-020; R&D Systems) were added. T cells were preactivated for 3 d before transfection.

Resting human CD4^+^ and CD8^+^ T cells were used for RNP transfection immediately after isolation.

Preactivated human CD4^+^ and CD8^+^ T cells were cultured at 1 × 10^6^ cells/ml of complete T cell media with 10 µg/ml plate-bound anti-CD3 (clone OKT3, catalog number 16-0037-85; eBioscience) and 1 µg/ml soluble anti-CD28 (CD28.2, catalog number 555725; BD Biosciences). For CD8^+^ T cells, 10 ng/ml recombinant human IL-2 (catalog number 202-IL-010; R&D Systems) was added. Cells were cultured for 3 d before transfection.

Human regulatory T cells were expanded for 7 d using the Human Treg Expansion kit (catalog number 130-095-353; Miltenyi Biotec) with 500 IU/ml recombinant human IL-2 (catalog number 130-097-746; Miltenyi Biotec).

#### Preparation of crRNA–tracrRNA duplex

To prepare the duplex, each Alt-R crRNA and Alt-R tracrRNA (catalog number 1072534; IDT) or Alt-tracrRNA-ATTO550 (catalog number 1075928; IDTd) was reconstituted to 100 µM with Nuclease-Free Duplex Buffer (IDT). Oligos were mixed at equimolar concentrations in a sterile PCR tube (e.g., 10 µl Alt-R crRNA and 10 µl Alt-R tracrRNA). Oligos were annealed by heating at 95°C for 5 min in PCR thermocycler and the mix was slowly cooled to room temperature.

#### Precomplexing of Cas9/RNP

In a PCR strip, three crRNA–tracrRNA duplexes (3 µl equal to 150 pmol each, total of 9 µl) and 6 µl (180 pmol) TrueCut Cas9 Protein v2 (catalog number A36499; Thermo Fisher Scientific) were gently mix by pipetting up and down and incubated at room temperature for at least 10 min.

#### Nucleofection

200 µl complete T cell media per well of a 96-well plate was prewarmed. 1–10 million T cells were resuspended in 20 µl primary cell nucleofection solution (P2 Primary Cell 4D-Nucleofector X kit S [32 RCT, V4XP-2032; Lonza] for human T cells and P4 Primary Cell 4D-Nucleofector X kit S [32 RCT, V4XP-4032; Lonza] for murine T cells). T cells were mixed and incubated with 15 µl RNP at room temperature for 2 min in round bottom 96-well plate. The cell/RNP mix was transferred to Nucleofection cuvette strips (4D-Nucleofector X kit S; Lonza). Cells were electroporated using a 4D nucleofector (4D-Nucleofector Core Unit: Lonza, AAF-1002B; 4D-Nucleofector X Unit: AAF-1002X; Lonza). Pulses for different T cell populations (DS137 for resting mouse CD4^+^ or CD8^+^ T cells, CM137 for activated murine CD4^+^ or CD8^+^ T cells, and EH100 for resting or activated human CD4^+^ or CD8^+^ T cells). After nucleofection, prewarmed T cell media was used to transfer transfected cells in 96-well plates. Resting human T cells were cultured at 1 × 10^6^ per well in 200 µl complete T cell media for 3–5 d. Mouse T cells were cultured with IL-7 as described above (Preparation of cells). See Table 1 for an overview of these conditions.

### Statistical analyses

GraphPad Prism software was used for an unpaired *t* test or one-way ANOVA with Dunnett’s multiple comparisons test to determine statistical significance.

### Online supplemental material

Fig. S1 shows low transduction and KO efficiency of retroviral sgRNA/Cas9 "all-in-one" constructs in primary CD4 T cells. Fig. S2 shows target gene KO with in vitro–transcribed sgRNAs in primary T cells. Fig. S3 shows CRISPR/Cas9-mediated KO of Foxp3 in expanded human regulatory T cells. Fig. S4 shows optimization of RNP transfection conditions in nonactivated mouse T cells. Table S1 lists nucleotide sequences of all sgRNAs and crRNAs.

## Supplementary Material

Supplemental Materials (PDF)

## References

[bib1] Chi S., Weiss A., and Wang H. 2016 A CRISPR-Based Toolbox for Studying T Cell Signal Transduction. BioMed Res. Int. 2016:5052369–10. 10.1155/2016/5052369 27057542 PMC4753324

[bib2] Chu V.T., Weber T., Graf R., Sommermann T., Petsch K., Sack U., Volchkov P., Rajewsky K., and Kühn R. 2016 Efficient generation of Rosa26 knock-in mice using CRISPR/Cas9 in C57BL/6 zygotes. BMC Biotechnol. 16:4 10.1186/s12896-016-0234-4 26772810 PMC4715285

[bib3] Cong L., Ran F.A., Cox D., Lin S., Barretto R., Habib N., Hsu P.D., Wu X., Jiang W., Marraffini L.A., and Zhang F. 2013 Multiplex genome engineering using CRISPR/Cas systems. Science. 339:819–823. 10.1126/science.1231143 23287718 PMC3795411

[bib4] Eyquem J., Mansilla-Soto J., Giavridis T., van der Stegen S.J.C., Hamieh M., Cunanan K.M., Odak A., Gönen M., and Sadelain M. 2017 Targeting a CAR to the TRAC locus with CRISPR/Cas9 enhances tumour rejection. Nature. 543:113–117. 10.1038/nature21405 28225754 PMC5558614

[bib5] Gomes-Silva D., Srinivasan M., Sharma S., Lee C.M., Wagner D.L., Davis T.H., Rouce R.H., Bao G., Brenner M.K., and Mamonkin M. 2017 CD7-edited T cells expressing a CD7-specific CAR for the therapy of T-cell malignancies. Blood. 130:285–296. 10.1182/blood-2017-01-761320 28539325 PMC5520470

[bib6] Hendel A., Bak R.O., Clark J.T., Kennedy A.B., Ryan D.E., Roy S., Steinfeld I., Lunstad B.D., Kaiser R.J., Wilkens A.B., 2015 Chemically modified guide RNAs enhance CRISPR-Cas genome editing in human primary cells. Nat. Biotechnol. 33:985–989. 10.1038/nbt.3290 26121415 PMC4729442

[bib7] Hsu P.D., Lander E.S., and Zhang F. 2014 Development and applications of CRISPR-Cas9 for genome engineering. Cell. 157:1262–1278. 10.1016/j.cell.2014.05.010 24906146 PMC4343198

[bib8] Jinek M., Chylinski K., Fonfara I., Hauer M., Doudna J.A., and Charpentier E. 2012 A programmable dual-RNA-guided DNA endonuclease in adaptive bacterial immunity. Science. 337:816–821. 10.1126/science.1225829 22745249 PMC6286148

[bib9] Kishimoto H., and Sprent J. 1999 Strong TCR ligation without costimulation causes rapid onset of Fas-dependent apoptosis of naive murine CD4+ T cells. J. Immunol. 163:1817–1826. 10438914

[bib10] Li C., Guan X., Du T., Jin W., Wu B., Liu Y., Wang P., Hu B., Griffin G.E., Shattock R.J., and Hu Q. 2015 Inhibition of HIV-1 infection of primary CD4+ T-cells by gene editing of CCR5 using adenovirus-delivered CRISPR/Cas9. J. Gen. Virol. 96:2381–2393. 10.1099/vir.0.000139 25854553

[bib11] Liu X., Zhang Y., Cheng C., Cheng A.W., Zhang X., Li N., Xia C., Wei X., Liu X., and Wang H. 2017 CRISPR-Cas9-mediated multiplex gene editing in CAR-T cells. Cell Res. 27:154–157. 10.1038/cr.2016.142 27910851 PMC5223227

[bib12] Mali P., Esvelt K.M., and Church G.M. 2013 Cas9 as a versatile tool for engineering biology. Nat. Methods. 10:957–963. 10.1038/nmeth.2649 24076990 PMC4051438

[bib13] Mandal P.K., Ferreira L.M.R., Collins R., Meissner T.B., Boutwell C.L., Friesen M., Vrbanac V., Garrison B.S., Stortchevoi A., Bryder D., 2014 Efficient ablation of genes in human hematopoietic stem and effector cells using CRISPR/Cas9. Cell Stem Cell. 15:643–652. 10.1016/j.stem.2014.10.004 25517468 PMC4269831

[bib14] Mantei A., Rutz S., Janke M., Kirchhoff D., Jung U., Patzel V., Vogel U., Rudel T., Andreou I., Weber M., and Scheffold A. 2008 siRNA stabilization prolongs gene knockdown in primary T lymphocytes. Eur. J. Immunol. 38:2616–2625. 10.1002/eji.200738075 18792414

[bib15] Platt R.J., Chen S., Zhou Y., Yim M.J., Swiech L., Kempton H.R., Dahlman J.E., Parnas O., Eisenhaure T.M., Jovanovic M., 2014 CRISPR-Cas9 knockin mice for genome editing and cancer modeling. Cell. 159:440–455. 10.1016/j.cell.2014.09.014 25263330 PMC4265475

[bib16] Ren J., and Zhao Y. 2017 Advancing chimeric antigen receptor T cell therapy with CRISPR/Cas9. Protein Cell. 8:634–643. 10.1007/s13238-017-0410-x 28434148 PMC5563282

[bib17] Ren J., Liu X., Fang C., Jiang S., June C.H., and Zhao Y. 2017 a Multiplex Genome Editing to Generate Universal CAR T Cells Resistant to PD1 Inhibition. Clin. Cancer Res. 23:2255–2266. 10.1158/1078-0432.CCR-16-1300 27815355 PMC5413401

[bib18] Ren J., Zhang X., Liu X., Fang C., Jiang S., June C.H., and Zhao Y. 2017 b A versatile system for rapid multiplex genome-edited CAR T cell generation. Oncotarget. 8:17002–17011. 28199983 10.18632/oncotarget.15218 PMC5370017

[bib19] Rupp L.J., Schumann K., Roybal K.T., Gate R.E., Ye C.J., Lim W.A., and Marson A. 2017 CRISPR/Cas9-mediated PD-1 disruption enhances anti-tumor efficacy of human chimeric antigen receptor T cells. Sci. Rep. 7:737 10.1038/s41598-017-00462-8 28389661 PMC5428439

[bib20] Rutz S., and Scheffold A. 2004 Towards in vivo application of RNA interference - new toys, old problems. Arthritis Res. Ther. 6:78–85. 10.1186/ar1168 15059269 PMC400443

[bib21] Schumann K., Lin S., Boyer E., Simeonov D.R., Subramaniam M., Gate R.E., Haliburton G.E., Ye C.J., Bluestone J.A., Doudna J.A., and Marson A. 2015 Generation of knock-in primary human T cells using Cas9 ribonucleoproteins. Proc. Natl. Acad. Sci. USA. 112:10437–10442. 10.1073/pnas.1512503112 26216948 PMC4547290

[bib22] Su S., Hu B., Shao J., Shen B., Du J., Du Y., Zhou J., Yu L., Zhang L., Chen F., 2016 CRISPR-Cas9 mediated efficient PD-1 disruption on human primary T cells from cancer patients. Sci. Rep. 6:20070 10.1038/srep20070 26818188 PMC4730182

[bib23] Tan J.T., Dudl E., LeRoy E., Murray R., Sprent J., Weinberg K.I., and Surh C.D. 2001 IL-7 is critical for homeostatic proliferation and survival of naive T cells. Proc. Natl. Acad. Sci. USA. 98:8732–8737. 10.1073/pnas.161126098 11447288 PMC37504

[bib24] Vella A., Teague T.K., Ihle J., Kappler J., and Marrack P. 1997 Interleukin 4 (IL-4) or IL-7 prevents the death of resting T cells: stat6 is probably not required for the effect of IL-4. J. Exp. Med. 186:325–330. 10.1084/jem.186.2.325 9221762 PMC2198981

[bib25] Wang W., Ye C., Liu J., Zhang D., Kimata J.T., and Zhou P. 2014 CCR5 gene disruption via lentiviral vectors expressing Cas9 and single guided RNA renders cells resistant to HIV-1 infection. PLoS One. 9:e115987 10.1371/journal.pone.0115987 25541967 PMC4277423

