# Supplementary material for: Optimized RNP transfection for highly efficient CRISPR/Cas9-mediated gene knockout in primary T cells
Source: J Exp Med. 2018 Mar 5;215(3):985–97. doi: 10.1084/jem.20171626 (PMC5839763; doi:10.1084/jem.20171626)
Supplement: Supplemental Materials (PDF) [file JEM_20171626_sm.pdf]

## SUPPLEMENTAL MATERIAL

Seki and Rutz, https://doi.org/10.1084/jem.20171626

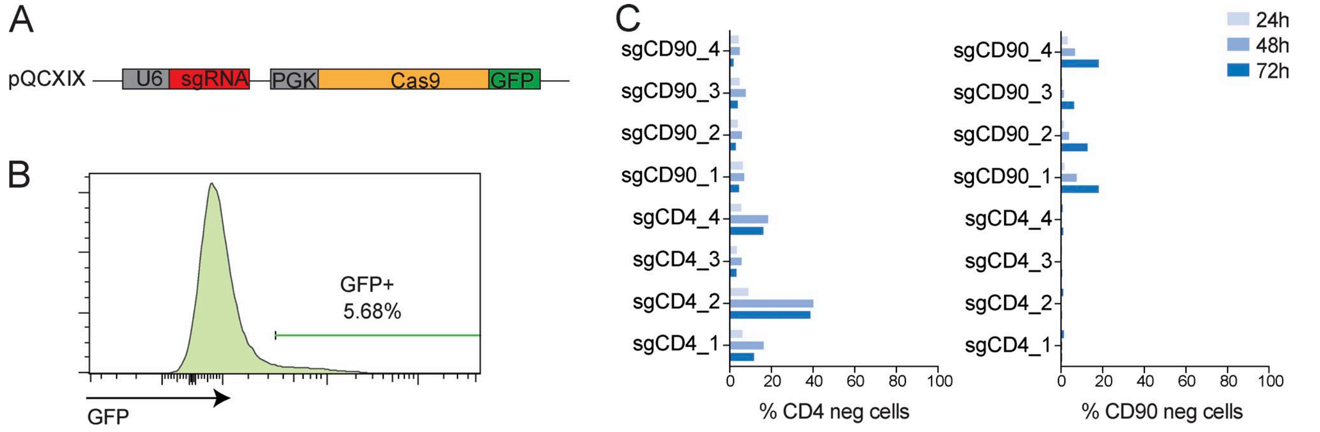


Figure S1. **Low efficiency of retroviral transduction of sgRNA/Cas9 all-in-one construct in primary CD4+ T cells. (A)** Schematic depiction of all-in-one retroviral construct expressing a sgRNA from a U6 polymerase III promoter and a Cas9/GFP fusion protein from a PGK promoter. **(B)** Expression of the retroviral construct as measured by GFP expression 48 h after retroviral transduction in CD4+ mouse T cells (gated on live cells). **(C)** Percentage CD4 or CD90 negative (KO) T cells 24, 48, and 72 h after retroviral transduction with sgRNA/Cas9 constructs targeting either CD4 or CD90 in CD4+ T cells (gated on live GFP+ cells). Data are representative of two independent experiments.

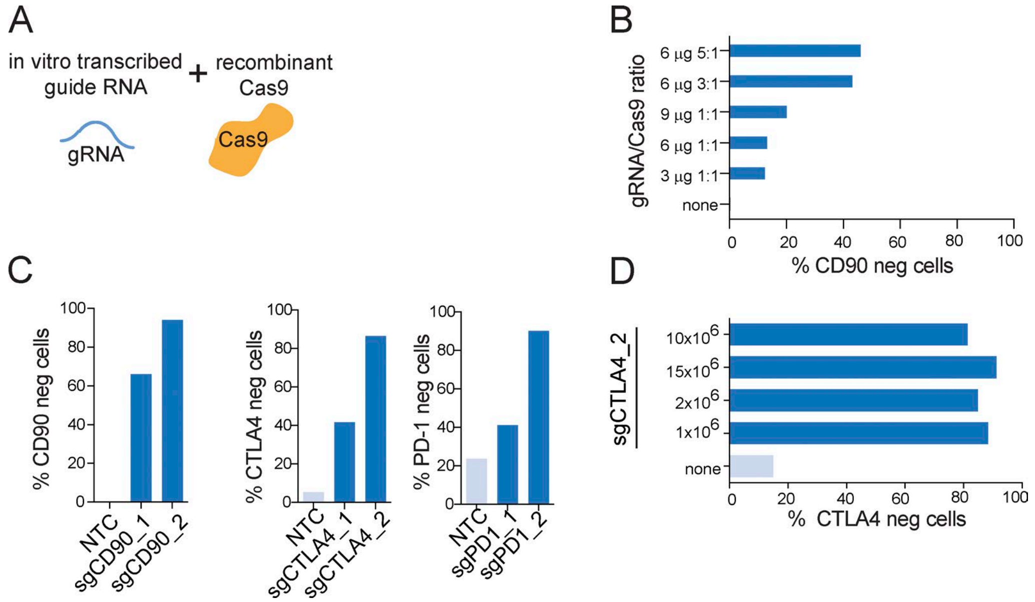


Figure S2. **Electroporation of in vitro transcribed sgRNA and recombinant Cas9 protein leads to highly efficient gene KO in primary T cells. (A)** Schematic depiction of RNP components, in vitro–transcribed sgRNA, and recombinant Cas9 protein. **(B)** Titration of amount and ratio of sgRNA/Cas9 targeting CD90 in activated mouse CD8+ T cells. Data are representative of two independent experiments. **(C)** KO efficiency as measured by flow cytometry 3 d after RNP transfection targeting CD90, CTLA4, and PD1 in activated mouse CD8+ T cells. Data are representative of two independent experiments. **(D)** Titration of number of activated mouse CD8+ T cells transfected with sgRNA targeting CD90. Data are representative of two independent experiments.

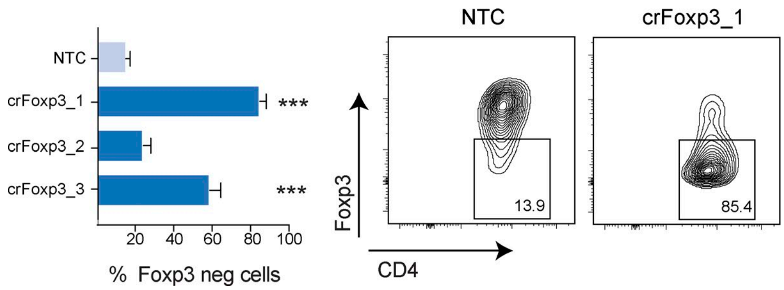


Figure S3. **KO of Foxp3 in expanded human regulatory T cells.** KO efficiency of targeting FoxP3 in human regulatory T cells expanded in vitro for 7 d was analyzed by flow cytometry 72 h after RNP transfection. Data are presented as mean ± SD ($n = 2$) and representative of two independent experiments. ***, $P < 0.001$ by one-way ANOVA.

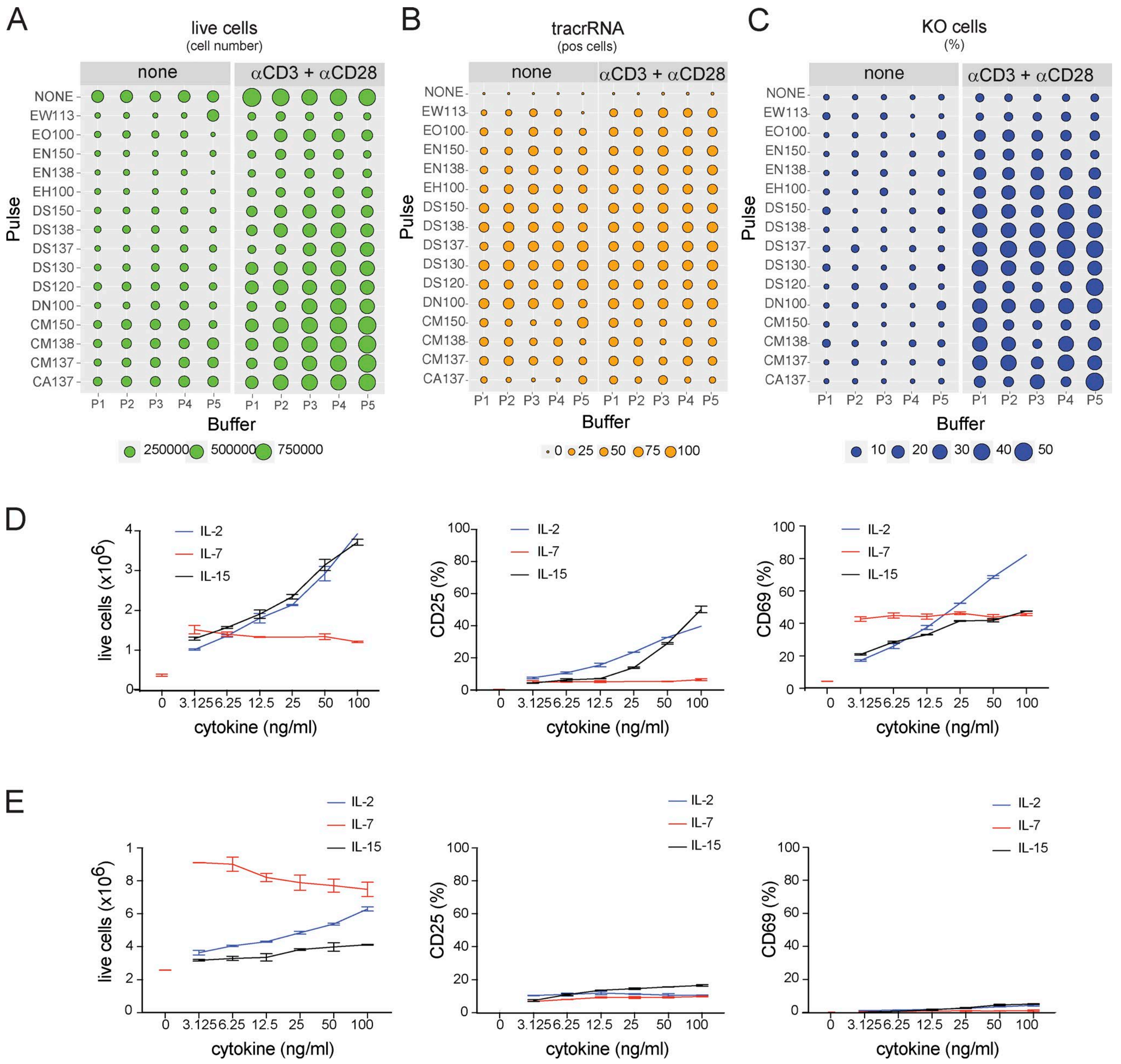

Figure S4. **Optimization of RNP transfection conditions in nonactivated mouse T cells. (A–C)** Systematic optimization of nucleofection parameters for RNP transfection of nonactivated mouse CD8+ T cells. Cell viability (A), transfection efficiency as determined by tracrRNA positive cells (B), and KO efficiency (%; C) of mouse CD8+ T cells transfected with RNP targeting CD90 and cultured for 2 d either without any stimulation or with anti-CD3/anti-CD28 stimulation. Data are from one experiment. **(D and E)** Analysis by flow cytometry of viable cells and CD25 and CD69 expression after 3 d of culture and titration of IL-2, IL-7, or IL-15 in CD8+ (D) or CD4+ mouse T cells (E). Data are presented as mean ± SD ($n = 2$) and representative of two independent experiments.

Table S1. **gRNA sequences**

| Name | Sequence (5′–3′) | Name | Sequence (5′–3′) |
|---|---|---|---|
| **sgRNA (plasmid constructs)** | | | |
| mCd4_1 | ACTCCTAGCTGTCACTCAA | mCd90_1 | ATGGCGGCAGTCCAGGCGA |
| mCd4_2 | AAGGGAAGACGCTGGTGCT | mCd90_2 | CTTGGTGTTATTCTCATGG |
| mCd4_3 | TAAGTTTATTGATGATGAG | mCd90_3 | TGCTGGATGGAGTTATCCT |
| mCd4_4 | AGAGTTGCTATCCAAGGTC | mCd90_4 | CTTCCTCTTCTCTCGGGTC |
| **sgRNA (IVT)** | | | |
| mCd90_1 | GGCGAAGGTTTTGGTTCACC | mCtla4_1 | GACCCAACCTTCAGTGGTGT |
| mCd90_2 | AAAGTAGTCGCCCTCATCCT | mCtla4_2 | ATGGAAAGCTGGCGACACCA |
| mPd1_1 | CGGAGGATCTTATGCTGAAC | | |
| mPd1_2 | GACACACGGCGCAATGACAG | | |
| **crRNA** | | | |
| mPd1_1 | ACAGCCCAAGTGAATGACCA | hCXCR4_1 | GAAGCGTGATGACAAAGAGG |
| mPd1_2 | TGAATGACCAGGGTACCTGC | hCXCR4_2 | AGGGAAGCGTGATGACAAAG |
| mPd1_3 | AGTTGAGCTGGCAATCAGGG | hCXCR4_3 | ACGGCATCAACTGCCCAGAA |
| mCtla4_1 | AGGTCCGGGTGACTGTGCTG | hCCR7_1 | TACCTACCTGCTCAACCTGG |
| mCtla4_2 | GACAAATGACCAAATGACTG | hCCR7_2 | CATCAGCATTGACCGCTACG |
| mCtla4_3 | ATGGAAAGCTGGCGACACCA | hCCR7_3 | AAAGTGGACACCGAAGACCC |
| mCd90_1 | CCGCCATGAGAATAACACCA | hCD127_1 | TCAGGCACTTTACCTCCACG |
| mCd90_2 | CCTTGGTGTTATTCTCATGG | hCD127_2 | CAGGCACTTTACCTCCACGA |
| mCd90_3 | GAGCAGGAGAGCGACGCTGA | hCD127_3 | CAAGTCGTTTCTGGAGAAAG |
| mCd8a_1 | ATCCCACAACAAGATAACGT | hIFNG_1 | CCAGAGCATCCAAAAGAGTG |
| mCd8a_2 | TGAAGCCATATAGACAACGA | hIFNG_2 | AAAGAGTGTGGAGACCATCA |
| mCd8a_3 | TGGGTGAGTCGATTATCCTG | hIFNG_3 | TGCAGGTCATTCAGATGTAG |
| mFoxp3_1 | TGGACGCACTTGGAGCACAG | hFOXP3_1 | CTTGAGGGAGAAGACCCCAG |
| mFoxp3_2 | TCTACCCACAGGGATCAATG | hFOXP3_2 | GAGGGAGAAGACCCCAGTGG |
| mFoxp3_3 | CTTGAGGGAGAAGACCCCAG | hFOXP3_3 | GGGGTGCACCTGCAGCACAG |
| | | hPD1_1 | CTGCAGCTTCTCCAACACAT |
| NTC | Negative control crRNA #1 (IDT 1072544) | hPD1_2 | CGTGTCACACAACTGCCCAA |
| | | hPD1_3 | GCCCTGGCCAGTCGTCTGGG |
| | | hCTLA4_1 | CCAGGTGACTGAAGTCTGTG |
| | | hCTLA4_2 | CACTGTCACCCGGACCTCAG |
| | | hCTLA4_3 | GCACGTGGCCCAGCCTGCTG |
| | | hTIGIT_1 | CAGGCACAATAGAAACAACG |
| | | hTIGIT_2 | GACCTGGGTCACTTGTGCCG |
| | | hTIGIT_3 | CAGCGCATGCTTCTGCCCAG |
